# Understanding Antibiotic Usage on Small-Scale Dairy Farms in the Indian States of Assam and Haryana Using a Mixed-Methods Approach—Outcomes and Challenges

**DOI:** 10.3390/antibiotics10091124

**Published:** 2021-09-18

**Authors:** Naresh Kumar, Garima Sharma, Eithne Leahy, Bibek R. Shome, Samiran Bandyopadhyay, Ram Pratim Deka, Rajeswari Shome, Tushar Kumar Dey, Johanna Frida Lindahl

**Affiliations:** 1 Dairy Microbiology Division, ICAR-National Dairy Research Institute, Karnal 132001, India; naresh.kumar2@icar.gov.in; 2 Department of Biosciences, International Livestock Research Institute, Nairobi 00100, Kenya; g.sharma@cgiar.org (G.S.); eithneleahy@hotmail.com (E.L.); r.deka@cgiar.org (R.P.D.); t.kumardey@cgiar.org (T.K.D.); 3 Department of Medical Biochemistry and Microbiology, Uppsala University, 75123 Uppsala, Sweden; 4 ICAR-National Institute of Veterinary Epidemiology and Disease Informatics, Bangalore 560064, India; bibek.shome@icar.gov.in (B.R.S.); rajeswari.shome@icar.gov.in (R.S.); 5 ICAR-Indian Veterinary Research Institute, Kolkata 700037, India; samiran.b@icar.gov.in; 6 Department of Clinical Sciences, Swedish University of Agricultural Sciences, 75007 Uppsala, Sweden

**Keywords:** antibiotic usage, smallholder dairy, India, KAP, residues in milk, farm inspection, farmer misconceptions

## Abstract

The use and misuse of antibiotics in both humans and animals contributes to the global emergence of antimicrobial resistant (AMR) bacteria, a threat to public health and infection control. Currently, India is the world’s leading milk producer but antibiotic usage within the dairy sector is poorly regulated. Little data exists reflecting how antibiotics are used on dairy farms, especially on small-scale dairy farms in India. To address this lack of data, a study was carried out on 491 small-scale dairy farms in two Indian states, Assam and Haryana, using a mixed method approach where farmers were interviewed, farms inspected for the presence of antibiotics and milk samples taken to determine antibiotic usage. Usage of antibiotics on farms appeared low only 10% (95% CI 8–13%) of farmers surveyed confirmed using antibiotics in their dairy herds during the last 12 months. Of the farms surveyed, only 8% (6–11%) had milk samples positive for antibiotic residues, namely from the novobiocin, macrolides, and sulphonamide classes of antibiotics. Of the farmers surveyed, only 2% (0.8–3%) had heard of the term “withdrawal period” and 53% (40–65%) failed to describe the term “antibiotic”. While this study clearly highlights a lack of understanding of antibiotics among small-scale dairy farmers, a potential factor in the emergence of AMR bacteria, it also shows that antibiotic usage on these farms is low and that the possible role these farmers play in AMR emergence may be overestimated.

## 1. Introduction

The emergence of antibiotic resistance in humans, animals, and the environment is strongly associated with the unnecessary use and misuse of antibiotics [1]. Antibiotic resistance is a global problem; however, low-and middle-income countries (LMICs) are considered most affected [2,3]. Globally, India ranks fourth in its usage of antibiotics in livestock [4]; however, regulations controlling antibiotic use in both human and animal medicine are very weakly enforced [5]. An understanding of the drivers and determinants of antibiotic usage at the farm level is lacking [6]. In higher-income countries (HICs), antimicrobial stewardship in veterinary medicine has gained increasing interest in recent years with extensive baseline data generated on antibiotic usage at farm level [6]. Management and practices relating to antibiotics on dairy farms in HICs are strictly controlled given the known transmission risk of antibiotic residues and antimicrobial resistant bacteria from dairy cows to humans [7]. India is a large and diverse country and evidence documenting antibiotic usage, especially on dairy farms, is lacking throughout the country [5,7]. In a country of this size and diversity, implementing controls on dairy farms is challenging [8]. However, with India standing as number one in the world for milk production [9,10], the challenge of generating this evidence regarding antibiotic usage at dairy farm level, is an urgent public health concern.

Across the milk value chain in India there is much geographical and institutional diversity, yet consistency is found in the size of farms; small-scale dairy producers predominating the milk supply chain [8]. It is estimated that some 70 million households derive their livelihoods from dairy cattle across India [11]. On these small-scale dairy farms, minimal quality control and infrastructure exists and practices, such as non-therapeutic or irrational use of antibiotics in lactating cows, are uncontrolled and unregulated [2,11,12]. Such malpractices do not seem as widespread on larger sized dairy farms which have been identified as using antibiotics in a more responsible way than smaller-scale dairy farms [1]. Overall, however, regardless of the size of the dairy farm, dairy farmers’ knowledge regarding antibiotics and reasons for antibiotic usage is poorly understood [2,13], and questions related to antibiotic practices, like quantity used, motive, administration method, frequency given, who administers, as well as adherence to withdrawal periods, remain poorly defined in the literature [11,14].

At a national level, work has been carried out within India to mitigate the emergence of antimicrobial resistance with the implementation of the 2017 National Health Policy and the revision of tolerance limits for the presence of antibiotics in foods of animal origin by the FSSAI (food safety and standards authority of India) [15]. The Food Safety and Standards (Contaminants, Toxins, and Residues) Regulations, 2011 [16], published by the FSSAI, has a thorough list of antibiotics/veterinary medications, and the indicated antibiotics must not exceed the tolerance limit provided for each food item, such as milk and meat. The Food Safety and Standards (Contaminants, Toxins, and Residues) Regulations, 2011 were changed on 1 August 2018 [17], to add new tolerance levels for 103 antibiotics and other veterinary medications in meat and meat products, poultry, fish, and milk. However, while these policy revisions act as guidelines from a top-down perspective on safe antibiotic usage, their uptake at a grassroots level remains poorly regulated and understood. Another study investigating farmers use of antibiotics on small-scale dairy farms in Assam and Haryana, through focus-group methods, showed poor levels of knowledge regarding antibiotic usage [12]. The use of other research methods including knowledge, attitudes, and practices (KAP) questionnaires among small-scale dairy farmers in Assam and Haryana has previously been reported, however the KAP research focused on zoonotic disease and not antibiotic usage [14,18,19]. Therefore, this study was designed with the objective of gaining an insight into antibiotic usage in small-scale dairy farms in North East India.

A mixed method, cross-sectional approach was used. A survey assessing farmer KAP regarding antibiotics, and their ability to recognize pictures of antibiotics, was combined with on-farm inspections and laboratory analysis of milk samples taken from the surveyed farms, to detect antibiotic residues. The study aimed to go beyond just assessing farmer knowledge levels. By investigating the farmer, the farm environment, and the product, milk, a broader, more cross validated, insight into antibiotic usage was hoped to be achieved. To the authors knowledge, this is the first study to use a three-pronged approach, investigating farmer, farm, and product combined to gain an overview of the role of small-scale dairy farms potentially play as hot spots for antimicrobial resistance emergence in northern India. It is hoped that the findings of this study will be used to inform future policy decisions and control strategies, such as the development of farmer-tailored intervention strategies, which may, over time, have an impact on the reduction of the emergence of antimicrobial resistance in the agricultural sector.

## 2. Results

Of the 491 farmers interviewed in this study, 84% were male (95% confidence interval (CI 80–87%) and 16% (CI 13–20%) of farmers were female. There were 42% (CI 38–47%) of farmers between 40 and 60 years of age, 38% (CI 34–43%) were between 20 and 40 years, 18% (CI 15–22%) were over 60 years of age and only 1% (CI 0.7–3%) were under 20 years of age. The most common education level of farmers was between 5–10 years of schooling. Farm size was determined by the number of milking bovines per herd; small scale farms had 1–3 milking bovines, medium scale farms had 4–9 and large-scale farms had >10 milking bovines. Of the 491 farms included in this study, 87% (CI 84–90%) constituted small scale, 9% (CI 7–12%) were medium and 4% (CI 3–6%) were large scale. Size of farm was found to be significantly associated with three of the ten variables recorded (Table 1).

### 2.1. Farmer Training

The farmer survey showed very low levels of training among farmers; large-scale farmers had received more training than small-scale farmers. Only 6% (CI 4–8%) of farmers surveyed had received training in livestock management. Of these, significantly more large-scale farmers (Table 1), 20% (CI 8–42%), had received training compared to 14% (CI 7–27%) of medium and 5% (CI 3–7%) of small-scale farmers. Only 3% (CI 2–5%) of farmers had received training in animal diseases: more large-scale farmers, 10% (CI 3–30%) received training in animal diseases compared to 2% (CI 0.2–12%) of medium-scale farmers or 3% (CI 2–5%) of small-scale farmers.

Neither training in livestock management nor animal diseases helped farmers’ ability to recognise an antibiotic. Only 45% (CI 28–62%) of farmers who completed the livestock training recognised antibiotics on picture cards. Of these, nine farmers, 66% (CI 47–80%) were small-scale farmers, 23% (CI 8–50%) were medium, and 15% (CI 4–42%) were large-scale farmers. Of the 17 farmers who had completed animal disease training, only 35% (CI 17–58%) were able to recognise antibiotic picture cards. Of these farmers, 82% (CI 59–93%) were small-scale farmers, 6% (CI 0.3–27%) were medium, and 12% (CI 3–34%) were large-scale farmers.

### 2.2. Knowledge and Use of Antibiotics

In total, 39% (CI 35–44%) of farmers responded that they had heard of antibiotics, with little variation between farm types; 40% (CI 22–61%) of large-scale farmers, 47% (CI 33–61%) of medium, and 39% (CI 34–43%) of small-scale farmers said they had heard of antibiotics.

Farmers were asked if they had used antibiotics in the last year on their farms, 10% (CI 8–13%) said yes, with significantly more antibiotic usage reported on larger and medium-scale farms compared to small-scale farms as seen in Table 1.

Only 2% (CI 0.8–3%) of the farmers surveyed had heard of the term “withdrawal period”. This was slightly more common among larger-scale farmers where 5% (CI 0.3–24%) reported to have heard of a withdrawal period compared to only 2.3% (CI 0.1–12%) of medium-scale farmers, and 1.4% (CI 0.6–3%) of small-scale farmers.

Of the 10% of farmers who responded that they had used antibiotics in the last 12 months, over half of these farmers, 53% (CI 40–65%), said they had never heard of antibiotics, yet supposedly they had used an antibiotic on their farm in the last year. Of the farmers who said they had used an antibiotic in the last 12 months but had never heard of antibiotics, 74% (CI 54–88%) were small-scale farmers, 13% (CI 5–32%) were medium, and 13% (CI 5–32%) were large-scale farmers.

### 2.3. Visual Recognition of Antibiotics

When shown picture cards with images of seven different antibiotics, only 11% (CI 9–14%) of all farmers were able to recognise images of antibiotics. Large-scale farmers, 30% of them (CI 15–52%), were significantly better able to recognise images compared to 19% (CI 10–32%) of medium-scale farmers, and 10% (CI 7–13%) of small-scale farmers (Table 1).

Farmer recognition of antibiotics on picture cards did not seem to correspond to farmer knowledge regarding antibiotics. Of the 11% (CI 9–14%) of farmers who recognised antibiotic picture cards, 47% (CI 35–60%) had responded in the questionnaire that they had never heard of antibiotics. Reasons for administering the recognized antibiotics to cattle included farmers perception of cows with a fever, mastitis, diarrhoea, or to increase milk production. Other reasons for treatment of cattle included liver problems, to increase appetite, to treat parasites, infections, wounds, joint pains, and ticks (listed as a separate issue to parasites by farmers).

### 2.4. On-Farm Inspection

Antibiotics were found and identified on 3% (CI 2–5%) of farms surveyed. More antibiotics were found on large scale farms, where 10% (CI 3–30%) of these farms were found to have antibiotics present compared to 5% (CI 1–16%) of medium-scale farms with antibiotic present and 2% (CI 1–4%) of small-scale farms found to have antibiotics present (Table 1).

On the 3% of farms where antibiotics were found on inspection, 57% (CI 33–78%), of these farmers had responded in the questionnaire that they had never heard of antibiotics, yet when shown antibiotic picture cards, 72% (CI 45–88%) of them had recognised at least one antibiotic on a picture card.

**Figure 1:**
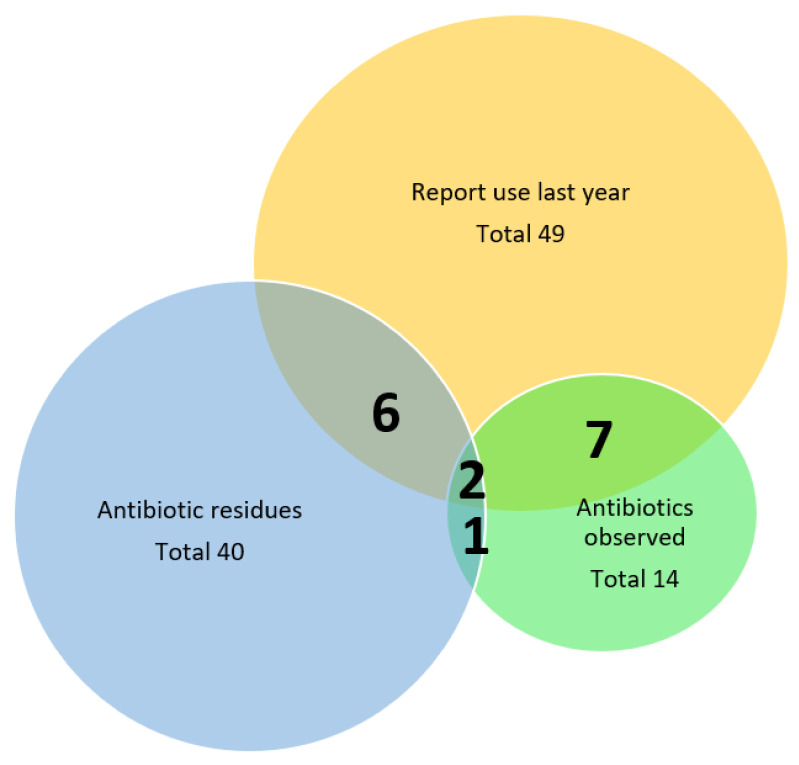
Venn diagram illustrating low correlation between farmer questionnaire responses, antibiotic residues found in milk samples, and antibiotics found on farms during farm inspections.

In total, 21 forms of antibiotic were found on farm inspections belonging to the following antibiotic groups: 14 beta-lactams, three fluroquinolones, two sulphonamides, and two aminoglycosides. Only three farms, 8% (CI 3–20%), where antibiotic residues were found in milk, had antibiotics present on farm inspection (Figure 1). These three farms were all small-scale farms. None of the antibiotic residues found in milk samples from these farms corresponded with any of the antibiotic products found on the same farms during inspection.

### 2.5. Milk Samples

In total, 40 samples from a total of 491, 8% (CI 6–11%) tested positive for antibiotic residues when screened with strip-based assay. Large-scale farms were found to have more positive samples with 20% (CI 8–42%) of these farms having residues compared to 12% (CI 5–25%) of medium and 7% (CI 5–10%) of small-scale farms (Table 1). Out of the 40 positive samples, 29 (5.9%, CI 4–8%) had levels above maximum residues limits (MRL) established by the European Commission of the European Union [20] (Table 2). Ten samples were positive above MRL for two antibiotics, and one sample was positive for three. All samples positive for tetracyclines were also positive for sulphonamides.

Figure 1 below illustrates how only eight of the 40 farms where residues were found in milk, corresponded to farmers who reported to have used antibiotics over the last 12 months in the KAP questionnaire.

Of the farms where antibiotic residues were found in milk, 73% (CI 57–84%) of farmers claimed not to have used antibiotics in the last 12 months, and out of these 63% (CI 31–86%) were small-scale famers, 25% (CI 7–60%) were medium-scale farmers, and 13% (CI 0.6–47%) were large-scale farmers.

It was found that 28% (CI 16–43%) of farmers from the farms where antibiotic residues were found in milk were able to recognize an antibiotic on picture cards. Of these, 64% (CI 35–84) were small-scale farmers while 18% (CI 5–48%) were medium, and 18% (CI 5–48%) were large-scale farmers.

Of the 40 farms where antibiotic residues were found in milk, not one farmer had heard of the term “withdrawal period”.

### 2.6. Veterinary Services

When asked about the frequency of veterinary visits to farms, 12% (CI 9–15%) of all farmers said they never received veterinary visits. Out of these, 97% (CI 88–99%) were small-scale farmers and the rest were medium-scale farmers. Similarly, low frequency of veterinary visits seemed higher in small farms than large-scale ones; of the 61% (CI 55–64%) of farmers who said they had veterinary visits less frequently than yearly, 87% (CI 83–90%) were small-scale farmers, 9% (CI 6–13%) were medium-scale farmers, and 4% (CI 2–6%) were large-scale farmers.

### 2.7. Multivariable Analysis

The alpha score calculated for the knowledge questions was 0.567, which is lower than the minimum acceptable value for Cronbach’s alpha (0.70), showing low consistency within the knowledge variables. The multivariable linear regression model showed a significant association between the knowledge alpha score with farm size and gender (*p* < 0.05). Female farmers and larger scale-farmers were likely to have good knowledge (high knowledge scores). No significant association between the knowledge alpha score and the state was found, see Table 3 below.

## 3. Discussion

A high degree of divergence was seen in this study between farmers’ knowledge, reported use of antibiotics, antibiotic presence on farms, and antibiotic residues in milk, making any predictions for antibiotic usage on these farms challenging. The KAP questionnaire results show that 39% of farmers claim to have heard about antibiotics, yet only 11% could positively identify an antibiotic on a picture card. Antibiotic usage on farms appears low, only 10% of farmers responded in the KAP questionnaire to have used an antibiotic in the last 12 months. However, of concern here is that 53% of those who said they had used an antibiotic had also responded that they had never heard of the word “antibiotic”. This raises the question of what do farmers understand by the term “antibiotic”? And, as a follow-on question to this, how much treatment of dairy cows occurs without the farmer understanding that he or she is using an antibiotic? Further conflicting results can be seen from the farm inspections; few farms were found to have antibiotics present on inspection (3% of farms) but where they were found, over half (57%) of these farmers had responded that they had never heard of antibiotics in the KAP questionnaire. When shown antibiotic picture cards, 72% of these same farmers, recognised an antibiotic picture card. Such results would indicate that farmer perception and visual recognition are at odds; farmers may recognise a picture card with an image of a commercially sold antibiotic without knowing or understanding what it truly is. This was further highlighted by the responses farmers gave as to why they used the medicine identified on the picture cards, responses included increasing milk production and treating parasites, neither of which are correct clinical reasons for antibiotic administration. Such poor consistency between farmer knowledge variables was statistically shown by the low Cronbach’s alpha score of 0.57.

In India, much has been discussed about the indiscriminate usage of antimicrobials in the Indian animal health sector and how it contributes immensely to antimicrobial resistance [1,2,11]. Indian dairy farmers have been targeted for their extensive and inappropriate antibiotic usage [8], their lack of observing withdrawal periods, and the consequential occurrence of antibiotic-contaminated milk [2]. Yet, our study points in a different direction. In total, only 3% of farms were found to have antibiotics present on inspection and only 8% of farms were found to have antibiotic residues in milk. Of the 40 out of 491 milk samples which showed some antibiotic residues present in milk, only 29 of these samples were deemed to be above maximum residues limits (MRL) established by the European Commission of the European Union. Therefore, in this study, less than 6% of our samples imposed a potential health risk to consumers. Such findings of so few milk samples exceeding the MRL have been reflected in other Indian studies [21]. Is it possible that the apportion of blame to small-scale dairy farmer as potential hubs of AMR emergence is exaggerated? Certainly, results from this study, showing both low levels of antibiotic usage and low levels of antibiotic residues in milk, would point towards these farms as low-input contributors to the bigger, national burden of AMR.

As per the Food Safety and Standards (Contaminants, Toxins, and Residues) Amendment Regulations, 2018 [17] some beta-lactam antibiotics, such as ampicillin and cloxacillin, have a tolerance limit of 0.01 mg/kg in milk; fluoroquinolones, such as enrofloxacin and danofloxacin, have tolerance limits of 0.01 mg/kg and 0.1 mg/kg, respectively; Sulfonamides such as sulfadiazine, sulfanilamide, and sulfathiazole sodium have a tolerance limit of 0.01 mg/kg, while sulfadimidine has a tolerance limit of 0.025 mg/kg, and the tolerance limit for aminoglycosides like streptomycin, dihydrostreptomycin in milk is 0.02 mg/kg, for lincomycin it is 0.15 mg/kg and for neomycin it is 1.5 mg/kg. For most antibiotics, the European Commission’s levels are similar or lower, and thus these were used for the study.

Our study found that none of the farmers from farms where antibiotic residues were found in milk samples had heard of a withdrawal period. Many studies have also observed that farmers do not observe withdrawal periods [2]. However, this topic merits further scrutiny. The results of our study show that it is not that farmers consciously disregard withdrawal periods, rather they are unfamiliar with the concept and seldom aware that the medicine they use is an antibiotic. This is an important distinction to make. While control and monitoring systems for residues in the dairy sectors of LMICs should be urgently implemented [4,5], such control systems must go hand in hand with improving local farmer knowledge to avoid jeopardizing the livelihood of small-scale dairy producers [3,22]. As evidenced in this study, residue-contaminated milk would have been due to a lack awareness, not a lack of compliance, and penalising these farmers would not mitigate future residue contamination. Findings like this emphasize the need for local milk production practices to be firstly investigated before effective control strategies can be implemented [11].

On the 40 farms where residues were found in milk, only 8% of these farms had antibiotics present on farm inspection. The antibiotic residues found in milk from these farms did not correspond with the antibiotic products found on these farms during inspection, suggestive of treatment by a visiting animal health personal without leaving the vial or prescription behind. However, another possible cause could be certain environmental factors [6]. Attributing blame for antibiotic residues exclusively to dairy farmers could be erroneous given that residues can be disseminated in the wider environment surrounding farms and their hinterlands [7]. This study highlights the importance of assessing local farmer knowledge as well as keeping in mind other factors, which can contribute to antibiotic residue-detection, before control measures are implemented. Future antibiotic residue testing should include local water supply samples, among others.

Despite overall poor levels of knowledge about antibiotics among the farmers in this study, we did identify some statistically significant differences between farmer groups; larger-scale farmers had an increased level of animal management training, increased ability to recognise antibiotic picture cards, and more often had used antibiotics in the last 12 months (Table 1). Future training interventions could target these cohorts of larger-scale farmers with tailor-suited interventions based on their current antibiotic usage and possible improved animal health awareness levels. Small-scale farmers were identified in this study as rarely or never receiving veterinary visits (Table 1) compared to large-scale farmers, yet small-scale farmers were found to have antibiotics present on farm inspection. This brings into question where and from whom are these farmers accessing antibiotics from, shedding light on other possible informal actors in the antibiotic supply chain visiting these farms that are not veterinarians [23]. The lack of veterinary prescriptions for antibiotics has been identified as a risk factor for antibiotic resistance in the dairy sector in India [1]. Therefore, these small-scale farmers make for a sub-cohort of particular interest and focus for future interventions in determining the source of non-prescribed veterinary antibiotics and gaining insight into informal medicine supply chains.

Including other stakeholders, such as animal health workers, paravets, or pharmacists, responsible for prescribing and dispensing antibiotics, often with limited knowledge surrounding antibiotics and resistance [13,23], was beyond the scope of this study. By focusing on only the farm level, this study informs only one piece of the larger scale antibiotic-resistance-puzzle; local veterinarians and pharmacists in Assam and Haryana have been identified as having poor awareness levels regarding antimicrobial resistance [8,13]. Therefore, including such stakeholders in future study designs is advisable, ignoring them creates an incomplete picture of antimicrobial use in livestock populations in LMICs [24].

Knowledge, attitudes, and practice (KAP) studies have been used extensively in low-resource countries, evaluating knowledge and the factors underlying the unsafe use of antibiotics but excluding visual recognition of antibiotics [25]. To avoid this common exclusion of visual recognition, our study attempted to combine a KAP questionnaire with visual aids in the form of antibiotic picture cards. These picture cards consisted of photographs of commercially sold livestock antibiotics taken from local pharmacies to represent medicines in the area. In this study, seven picture cards were shown to farmers, farmers were firstly asked if they recognised the product on the picture card, and secondly if they could name it. The use of visual aids has proved effective in determining correct antibiotic usage in communities with low levels of literacy in other lower income settings [26]. However, the method of picture card recognition used in this study, did not determine a farmer’s ability to recognise a product as an antibiotic per se, rather they were simply asked if they recognised it as a product, yes or no. This flaw in the study design meant that determining if a farmer can distinguish, using visual aids, an antibiotic from any another animal health related product, such as an anthelmintic for example, was not assessed. Despite limitations in our study, the results form a preliminary insight into potential challenges future policy decision makers may face when designing, and implementing, antibiotic control and regulation strategies. This study is the first of its kind, to date, to show how a mixed method approach can reveal complex challenges when it comes to understanding small scale dairy farmer antibiotic usage.

## 4. Conclusions

Irrational use of antibiotics in Indian dairy systems is aggravated by a number of factors; poor knowledge and misconceptions about antibiotics, easy access to antibiotics, and limited field supervision, possibly due to inadequate veterinary coverage [12], all seen in our study. Despite the three-pronged approach used to assess farmer, farm environment, and farm product, milk, quantifying antibiotic usage was hugely challenging in this study with farmers misconceptions on what antibiotics are remaining a fundamental stumbling block in quantifying antibiotic usage. Conflicting results between farmer KAP questionnaire, picture card identification, farm inspection, and milk residue testing show that no one method should be used as a stand-alone measure of antibiotic usage on these small dairy farms. Low usage of antibiotics by these small-scale dairy farmers plus low levels of antibiotic residues in milk samples are positive outcomes from the study, indicating the more minor role these farmers play in the emergence of AMR.

In LMIC’s, it has been suggested that investigating associations between antibiotic usage and antimicrobial resistance can only be achieved by measuring antibiotic usage in animal production, monitoring it over time and promoting reductions in antibiotic usage [5]. From our findings these objectives are highly unrealistic and problematic; establishing a baseline for antibiotic usage alone is already very challenging, not to mention introducing a monitoring process. That said, the importance of milk production for food security and nutrition [4,24,27] and the growing number of livelihoods relying on the dairy sector across India, means that these challenges must be confronted sooner rather than later if safe and sustainable dairy development is to continue.

## 5. Materials and Methods

### 5.1. Sampling Design

**Figure 2:**
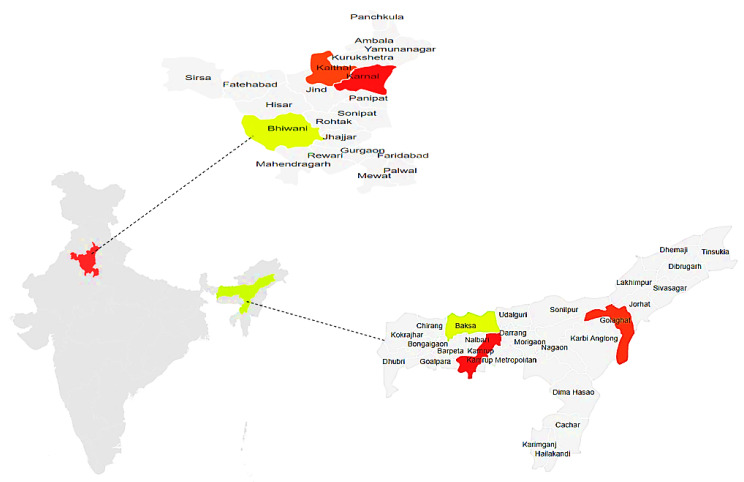
Map of India showing Haryana and Assam with the three districts within each state used for the study.

The cross-sectional study was conducted in the states of Assam and Haryana, in Northeast India from April 2016 to March 2017, using a multistage sampling technique for household sample selection in both states. These states were chosen based on their highly divergent situation in dairy production, with Haryana having the benefit of closeness to the Delhi market, while Assam, as one of the north-eastern states, is lagging behind in dairy development [28]. The first stage was to select three districts in each state. District selection was guided by Animal Husbandry and Veterinary Department officials regarding the district’s potential for dairy development (low, medium, and high). Accordingly, the three districts Kamrup (metropolitan), Golaghat, and Baksa were selected for Assam and Karnal, Kaithal and Bhiwani for Haryana. Figure 2 below shows a map of the two states with the three respective districts per state where the study was conducted. From each district, two community development blocks (CDBs) were selected, in Haryana one urban and one rural CDB were selected while in Assam, one urban and one peri-urban/rural CDB were selected. Then, four villages were selected randomly within each CDB, in the case of Kamrup Metropolitan district, where both CDB were more urban in nature, villages were defined as clusters of milk producers. Ten households were selected randomly from the list of farming households having dairy animals (cattle or buffalo) in each selected village or cluster. Household selection was guided by key informants such as local non-governmental organizations and some leading farmers, including village leaders. In total, 242 dairy farming households in Assam and 249 households were selected in the Haryana. Random selection was done using the random number function in MS Excel.

### 5.2. Ethics Statement

Ethical approval for the study was granted by the Institutional Research Ethics Committee (IREC) of the International Livestock Research Institute (ILRI) on 21 September 2015 (No. ILRI-IREC2015-12).

### 5.3. Data Collection

Selected farmers were contacted the day before farm visits by key informants. Farmers were asked to keep a sample of milk from the following mornings’ milking. At the time of visit, signed, informed consent was obtained from all farmers. Knowledge on antibiotics and their usage was assessed through a farmer questionnaire (Supplementary Materials). In addition, seven different picture cards were shown to farmers to assess farmers ability to recognise these products. Each card had multiple images of locally available antibiotics, within the same antibiotic family. Following the completion of the farmer interview, a farm inspection was carried out by enumerators and the presence of all animal health related products on farms recorded.

A bulk tank milk sample was taken from all farms, and where a bulk tank sample was not available, individual cows were milked. All samples were collected in sterile containers, transported in cold boxes to the laboratory and kept at −20 °C until analysis.

### 5.4. Analysis of Antibiotic Residues

Screening of milk samples for antibiotic residues was conducted in two steps. First two semi-quantitative tests were used, dipicolinic acid (DPA) test and a strip test, developed by National Dairy Research Institute (ICAR-NDRI), Karnal, India, was used. Positive results were further tested with the Charm Rosa method, Rapid One Step Assay lateral flow strips (Charm Sciences Inc., Lawrence, MA, USA) to identify which antibiotic groups were present, and to evaluate if samples exceeded the maximum residue levels set by the European Commission of the European Union [20].

### 5.5. Antibiotic Residue Analysis by Dipicolinic Acid (DPA) and Strip Test

The DPA test is a test based on the spore germination principle. The transformation of dormant *Bacillus stearothermophilus* spores into active vegetative cells is used to detect the absence of antibiotics [29]. As a unique strategy for detecting target pollutants in milk, the suppression of the germination process specifically in the presence of antibiotic residues was applied. The indicator organism, i.e., *B. stearothermophilus*, was initially allowed to sporulate by seeding in sporulation medium and incubating at 55 °C for 48 ± 2 h. It took 3 h for germination and outgrowth in minimal medium inoculated with activated spores, with dextrose, whey powder, skimmed milk powder, and reconstituted milk as a negative control. For the performance of this test, 100 µL of milk is added to the test tube. Antibiotic residues in milk were detected by changing the medium’s color from purple to yellow [30,31]. The test has been developed at ICAR-NDRI and patented (Patent no. 264145).

The strip test was developed by functionalizing spores and specific enzyme substrate. Here a paper strip is dipped in milk and then added to a tube with a disc containing nutrient for spore germination on strip and incubated at 64 °C for 75 min. Results were interpreted depending on color; blue color indicating absence of antibiotics while no color indicates presence of antibiotics at or above MRL. The test has been developed at ICAR-NDRI with patent no 2213/DEL/2014.

Samples positive with DPA and strip test were further analysed by Charm Rapid One Step Assay (ROSA) test, to identify which antibiotic group the residues belonged to. For statistical analyses, all samples positive with DPA and strip test were considered positive, irrespective of antibiotic class.

### 5.6. Quantitative Charm ROSA (Rapid One Step Assay) Test

The Charm ROSA test is a multiplex immuno-receptor lateral flow test that can detect several antibiotic residues simultaneously [32]. Charm ROSA antibiotic strips were procured from Charm Sciences Inc. (Lawrence, MA, USA). Milk samples identified as positive for antimicrobial residues by spore-based tests were analysed with ROSA test for detection different antibiotics and antibiotic groups: β-lactams (LF-MRLBL-100K), chloramphenicol (LF-CAP-100K), tetracycline (LF-TET-100K), sulphonamides (LF-SULFMRL-100K), streptomycin (LF-STREP-100K), gentamycin (LF-GENTA-100K), quinolones (LF-QUIN-100K), macrolides (MMRL-100K), novobiocin (NTBL-100K), and enrofloxacin (LF-ENRO-100K), antibiotics are commonly used in the treatment of cattle.

### 5.7. Statistical Analysis

Data was entered in Microsoft Excel and analysed using ‘R’ statistical software [33] and STATA 14.0 (STATACorp Ltd., College Station, TX, USA).

Descriptive data for all farms was combined with laboratory results corresponding with those farms milk samples. Initial univariable analyses were conducted using Fisher’s Exact Test for associations between categorical variables and farm size. A score was allotted to farmers’ responses in the questionnaire. A score of ‘1′ was assigned to a correct answer and a ‘0′ score assigned for the wrong answers. The total mean score was then calculated for each respondent. Internal consistency was measured using Cronbach alpha. A Venn diagram was used to illustrate lack of correlation between three variables. A multivariable linear regression model was built in STATA to test correlation between the alpha knowledge score with state, gender, and farm size. The map was prepared using Gramener-India map (https://gramener.com/indiamap/) (accessed on 28 May 2021).

## Figures and Tables

**Table 1:** Dairy farmer training and knowledge about antibiotics in Assam and Haryana, India.

	Total Farmers	Total % (CI ^$^)	Small-Scale Farm% (CI)	Medium-Scale Farm% (CI)	Large-Scale Farm% (CI)	*p*-Value ^#^
Completed training in animal management	29/491	6% (4–8%)	4.5% (3–7%) *	14% (7–27%)	20% (8–42%) *	0.002
Completed training in animal disease	17/491	4% (2–6%)	3% (2–5%)	2% (0.2–12%)	10% (3–30%)	0.195
Farmers who recognise antibiotics on picture cards	55/491	11% (9–14%)	10% (7–13%) *	19% (10–32%)	30% (15–52%) *	0.006
Farmers who say they have heard of antibiotics	193/491	39% (35–44%)	39% (34–43%)	47% (33–61%)	40% (22–61%)	0.591
Farmers who say they have heard of a withdrawal period	8/491	2% (0.8–3%)	1.4% (0.6–3)	2.3% (0.1–12)	5% (0.3–24)	0.180
Farmers who say they used antibiotics in last year on their farm	49/491	10% (8–13%)	9% (6–12%) *	16% (8–30%)	25% (11–47%) *	0.021
Farms where antibiotic residues were found in milk	40/491	8% (6–11%)	7% (5–10)	12% (5–25%)	20% (8–42%)	0.076
Farms where antibiotics were found on inspection	14/491	3% (2–5%)	2% (1–4%)	5% (1–16%)	10% (3–30%)	0.068
Farms who report veterinary visits yearly or less	292/491	61% (55–64%)	87% (83–90%)	9% (6–13%)	4% (2–6%)	0.827
Farms who report never having a veterinary visit	58/491	12% (9–15%)	97% (88–99%)	3% (1–12%)	0%	0.07

* Significantly different within the row at *p* < 0.05, ^#^ Fisher’s exact test, ^$^ 95% confidence interval (CI).

**Table 2:** Maximum residue levels (MRL) of the European Commission for antibiotic residues in milk, and the number of milk samples exceeding this.

Antibiotic/Antibiotic Group	MRL in Milk (European Commission)	Number of Samples Exceeding MRL
Beta-lactam antibiotics (penicillin)	4 µg/kg	3 (0.6%)
Tetracycline/chlortetracycline/oxytetracycline	100 µg/kg	4 (0.8%)
Sulphonamides	100 µg/kg	5 (1.0%)
Streptomycin/Dihydrostreptomycin	200 µg/kg	0
Gentamicin	100 µg/kg	0
Novobiocin	50 µg/kg	17 (3.5%)
Macrolides (Erythromycin)	40 µg/kg	12 (2.4%)
Quinolones (Enrofloxacin)	100 µg/kg	0
Chloramphenicol	Prohibited	0

**Table 3:** Multivariable regression results of knowledge score, normalized with Cronbach’s alpha, with farm size, state, and gender.

	Coefficients	Standard Error	*p*-Value	95% CI
**Farm Size**				
Small farms	Reference category			
Medium farms	0.07	0.03	0.054	0.001–0.14
Large farms	0.08	0.05	0.050	0.01–0.18
**State**				
Assam	Reference category			
Haryana	0.03	0.02	0.19	0.01–0.07
**Gender**				
Male	Reference category			
Female	0.11	0.03	<0.001	0.17–0.05

## Data Availability

Data is made available from the authors upon reasonable request.

## Supplementary Materials

The following are available online: https://www.mdpi.com/article/10.3390/antibiotics10091124/s1. Farmer questionnaire.
