# Supplementary material for: Understanding Antibiotic Usage on Small-Scale Dairy Farms in the Indian States of Assam and Haryana Using a Mixed-Methods Approach—Outcomes and Challenges
Source: Antibiotics (Basel). 2021 Sep 18;10(9):1124. doi: 10.3390/antibiotics10091124 (PMC8468593; doi:10.3390/antibiotics10091124)
Supplement: Supplementary file 1 [file antibiotics-10-01124-s001.zip › antibiotics-1321299-supplementary.pdf]

Questionnaire number:

Questionnaire

1. Name and address of the respondent NB: must be responsible for bovine health decisions

1.1	Name of interviewer		1.2	Date	
1.3	North		1.4	East	
1.5	Name of the HH head		1.6	Phone no.	
1.7	Village/Ward		1.8	Block/Town	

2. Basic demography & farm details

2.1	Gender of respondent (1=male, 2=female)		2.2	No of HH members	
2.3	Highest education (0= No education, 1=primary, 2=class 5-10, 3=Higher secondary, 4=Graduation and above)		2.4	Age (years)	

2.5	Have any member of your family ever availed training on livestock management (Yes=1, No=0)	
2.6	Did you ever receive training on animal disease? (1=Yes, 0=no)	
2.6.1	If yes, who gave it?	
2.6.2	If yes, did you learn about giving medicines to animals?	
2.6.3	Do you hand milk or use machine? (Hand milking =1, machine milking =2)	

3. Herd size

Sl. No.	Species	milking	dry cows/ buffalo cows	adult bulls/bullock	heifer	Calves
3.1	Cattle					
3.2	Buffalo					

4. Picture card

		Product a	Product b	Product c	Product d	Product e
4.1	Do you recognize this medicine? (1=Yes, 0=no)					
4.2	What is its name?					
4.3	Did your cows have this medicine in the last 12 months?					
4.4	For which reason was it used?					

5.Knowledge and practice on antibiotic use

5.1	Have you heard about antibiotics? (1=Yes, 0=no)	
5.2	**If yes,** Please describe what antibiotics do:	
5.3	Have you heard of withdrawal period (1=Yes, 0=no)	

Questionnaire number:

5.4	**<u>If yes,</u>** Please describe it :	
5.5	When do you stop treating an animal with a medicine?<br>(1= when I see it is recovering OR looks healthy<br>2= Continue as long as veterinary says 3= When I don't have more money/medicine to treat)	
5.6	Where are medicines discarded?<br>(0= throw away outside 1= garbage bin 2= Return to seller 3= burn 4= Bury<br>5= drain 6= don't bother 7= others:___________)	

6. Knowledge and attitude towards antibiotic use

	Do you agree with the following:<br>If farmer has not heard about antibiotics use medicine instead of antibiotic	agree=1,/<br>disagree=0/<br>Don't know=99
6.1	The more an antibiotic / medicine costs, the better it is	
6.2	All antibiotic/medicine suitable for people can be used in animals	
6.3	If the milk from a treated cow looks normal it is ok to consume	
6.4	The pharmacist is as good as the vet to decide which antibiotic/medicine	
6.5	All diseases need treatment	
6.6	Injections are always more powerful than oral antibiotic/medicine	
6.7	There are many poor quality antibiotic/medicine in the market	
6.8	Increasing the amount of antibiotic/medicine will make it more effective	
6.9	Antibiotics stop all diseases	
6.10	If one cow is sick, then others should be treated also to prevent disease	
6.11	Antibiotics can cure foot and mouth disease	
6.12	If using antibiotic too often, it may stop being effective	

7. Medical treatments

<table>
<tr><td colspan="2">7.1 When you treat your animals with medicines, what do you do with the milk during those days?
1. Sell 2. Throw away 3. Keep in the household for consumption
4. Offer to calves 5. Others, specify: ____________________________</td><td></td></tr>
<tr><td colspan="2">7.2 What do you do with the milk of a sick animal which is not treated?
1. Sell 2. Throw away 3. Keep in the household for consumption 4. Offer to calves 5. Others, specify: _____________________________</td><td></td></tr>
<tr><td>7.3 How often do you have veterinary consultations?</td><td>0=Never, 1= weekly (more than once per week), 2=monthly (more than once per month), 3= yearly, 4= more seldom</td><td></td></tr>
</table>

Questionnaire number:

8 Cattle illness in the preceding 12 months

Sl.No	Disease/ symptom/ condition	How many animals got this last 12 months? **99= don't know, 0= No animals**	Trend in last 12 months from previous years **1= More this year; 2= less this year; 3= no change**	How many animals got treated with medicine last 12 months? **99= don't know, 0= No animals**	Name of medicine or type of medicine given for this last time cattle got sick.
8.1	Mastitis				
8.2	Respiratory				
8.3	Diarrhea				
8.4	Lameness				
8.5	Fever				
8.6	abnormal vaginal discharge (pus, strange color or odour)				
8.7	Repeat breeding				
8.8	Abortion				
8.9	Retention of placenta				
8.10	Still birth				
8.11	Male infertility				
8.12	Carpal hygroma				
8.13	Any other specific disease you want to mention:				
	8.14 Did any cow have an allergic reaction after treatment? (1=Yes, 0=no)<br>8.15 **If yes,** How many last 12 months?				

Questionnaire number:

Observation check list

9 Farming system details

	Parameters	Response
9.1	Floor type (0=Bricks, 1=concrete, 2= Earthen, 3= other (specify))	
9.2	Cleanliness of farm house (1 = very, 2 =some, 3 = dirty)	
9.3	Roof: 1= Pakka/concrete slab 2= Thatched roof 3= corrugated tin/asbestos, 4= traditional roofing tiles, 5= concrete tiles, 6= bamboo corrugated sheet 7= other…………………	
9.4	Separate houses for: 1= calves, 2= Pregnant, 3= Sick (Multiple options allowed)	
9.5	Cleanliness of cows (1=very, 2=some, 3=not)	
9.6	Medicine storage (0= no storage 1=Open and used packages stored, 2=sealed cabinet, 3=exposed to sun, 4= nothing specific, 5= store house/cattle shed) Multiple options allowed	
9.7	Are there discarded medicines, wrappers, syringes on farm premises (1=yes, 0=no)	

10. Medicine list: **Make a list of all the medicines on the farm including any medicated food and discarded packages Containing antibiotics. If unsure, list it.**

Medicine name	Quantity	Expiry date	open/closed package	What farmer uses for	How much used in last 12 months	Where or from whom did you buy this pack?
